# Intended cycling frequency and the role of happiness and environmental friendliness after COVID-19

**DOI:** 10.1038/s41598-023-27681-6

**Published:** 2023-01-12

**Authors:** Natalia Barbour, Fred Mannering

**Affiliations:** 1 Department of Civil, Environmental & Construction Engineering, University of Central Florida, Orlando, FL 32816 USA; 2 Department of Civil and Environmental Engineering, University of South Florida, 4202 E. Fowler Avenue, ENB118, Tampa, FL 33620 USA

**Keywords:** Civil engineering, Psychology and behaviour

## Abstract

Although the COVID-19 pandemic has contributed to an increase in cycling in many countries worldwide, it is not yet known whether this increase becomes a long-lasting change in mobility. The current study explores this increase by analyzing data collected in a U.S. nationwide longitudinal survey. Using a total of 7421 observations, a mixed logit model with heterogeneity in the means of random parameters was estimated. In the resulting sample, nearly 14 percent of the respondents stated that they were planning to cycle more while only 4 percent of the respondents stated that they were planning to cycle less post COVID-19 pandemic. The estimation results provide insights into socio-demographic and psychological factors that play a role in planned cycling behavior post COVID-19. The study also establishes that age, race, employment status, gender, and household size impact intended cycling frequency. The model estimation results further indicate that workers (full time and part time), individuals with a high degree of life satisfaction, and individuals who are environmentally friendly all have higher cycling-frequency probabilities relative to others. The findings can be used to support policies that target sustainable mobility and further our understanding of the transportation, psychology, and well-being relationships.

## Introduction

Transportation is no longer just about moving large numbers of cars in the shortest amount of time or improving the efficiency of public transit systems—the focal point of discussions have turned to equity, health, access, and social justice. Broadening the transportation landscape by considering additional modes and dimensions that are found in urban design, health, and psychology recognizes the importance that transportation systems play in daily lives of people worldwide^1^. The need to rethink the composition of the sector, its priorities, operations, and performance has been magnified by the two global events: the ongoing climate crisis and the COVID-19 pandemic. Transportation has long been one of the main contributing sectors to climate change, in the United States it accounts for approximately 30 percent of greenhouse gasses^2,3^. While transportation-related emissions and the need to decarbonize the sector are central to achieving Paris Agreement goals^4,5^, the COVID-19 pandemic has been a major disruptor, fundamentally changing how the transport system is perceived, affecting daily commutes, and encouraging many users to try alternate, and often non-motorized, transportation modes^6–9^.

In recent years, the shift to cycling has gained a substantial attention in research and practice^6^. Even before the pandemic, research pointed to the many virtues of non-motorized options like walking, cycling, and other micromobility modes as options to increase transport efficiency and reduce emissions^5,10^ Buehler and Pucher^6^ examined these trends during the pandemic over time and by location, and concluded that there had been considerable variation in the percentage changes in cycling levels between 2019 and 2020 among EU countries as well as among regions in the U.S. By comparing both full years, including periods of lockdown in 2020, they found that the 11 EU countries averaged an overall 8 percent increase in cycling while the U.S. averaged 16 percent increase.

Research that addresses cycling and explores it in the context of public gains has found its benefits to include cost savings, savings on journey time, convenience, health, and perceived utility benefits to well-being^11–14^. Benefits also include improvements in the livability of cities and public health^15^. From an environmental perspective, numerous studies have highlighted the potential for cycling to reduce motor vehicle use and the associated external social costs that are imposed in terms of pollution, greenhouse gases, dirt, noise, and congestion^14,16^.

Past work has also stressed the relationship between cycling and the design of the active mobility infrastructure, and the ways in which transportation planning, policy, behavioral economics, and engineering fields might advance to best support the shift from auto-centric to human-centric designs^7,17^. With observed shifts toward human-centric transport, the growing need for human-centric designs and how they might support and encourage such shifts has begun to be explored in the literature^6,18,19^. However, comparatively little is known about the specifics responsible for human-centric shifts or how the underlying dynamics will affect potential changes in the future^18^. Studies of travel behavior in context of COVID-19 pandemic, which have to varying degrees explored shifts in cycling behavior, have found that factors such as age, education, gender, employment, household size, and car ownership were influential in determining cycling use^17,19^.

With the cycling revolution reflected in the increased use and advocacy of this transport mode^20^ there is acknowledgement that understanding cycling use and trends is a multifaceted problem influenced by global perspectives, preferences, and attitudes. For example, studies have started to explore the relationship between environmental friendliness, health as well as lifestyle and the propensity to cycle, with many concluding a clear connection between psychological and lifestyle related variables and the willingness to cycle^21–23^. Over the years, the impacts of physical infrastructure on cycling have been well documented and, in addition, the impacts of attitudes and social norms have been recognized as important considerations^24,25^. For example, previous studies have highlighted the fact that significant changes in transport-related attitudes, norms, and behaviors have the potential to result in immediate reductions in fossil fuel consumption, and establish the foundations for further policy, public investment, and industry shifts^26,27^. Work by Wang et al.^28^ found that attitudes towards greenness and environmental concerns were significantly and positively correlated to cycling adoption intentions. Past research has also confirmed the substantial impact of environmental concerns on various behaviors^29,30^.

Growing societal awareness about climate change mitigation, coupled with a worldwide encouragement for sustainable behaviors, have been identified as factors influencing the public opinions about cycling. The evolution of public opinions towards cycling was underscored by Willis et al.^31^ who argued that social factors clearly affect the decision to cycle, and it is essential to look beyond the role of physical and built environment factors when attempting to understand or predict use.

Especially in the context of intended cycling post COVID-19 pandemic, social and psychological factors, and their role must be considered to gain substantive insights. Because pandemic related measures were often dictated by the government, the modifications to one’s mobility did not necessarily arise primarily from free will, but instead were a response to various externally imposed constraints, resulting in many people trying modes and behaviors they would not have tried otherwise^32^.

The current work applies an advanced econometric modeling approach to study the effect of specific socio-demographic characteristics of cyclers who plan to change their cycling frequency after the pandemic. The analysis does not only examine the evolution of cycling frequency during the COVID-19 pandemic (which is particularly important when considering the need to reduce emissions) but also links human well-being, life satisfaction, and environmental friendliness to intended cycling frequency. Thus, the empirical work herein moves beyond the traditional set of socio-demographic variables to explore the relationship between lifestyle and psychological factors and the role they play in the growth in sustainable mobility. The aim of the current study is to provide a better understanding of the shifts toward cycling and relate these shifts to policy recommendations.

## Data and methods

The publicly available data used for the forthcoming analysis are from a survey that was a joint project of Arizona State University and the University of Illinois Chicago with support from the National Science Foundation. The survey includes responses from the adult population of United States citizens and was conducted using a nationwide longitudinal questionnaire that gathered information about travel-related behaviors and attitudes before, during, and after the COVID-19 pandemic. To capture shifts in behavior the survey was divided into waves that gathered information at different phases of the pandemic. In addition, the data were weighted to be representative of national and regional demographics. The survey questions covered a wide range of topics including commuting, daily travel, air travel, working from home, online learning, shopping, and risk perception, along with attitudinal, socioeconomic, and demographic information.

The survey was deployed over multiple waves to the same respondents to monitor how behaviors and attitudes evolved over time. The questionnaire was disseminated digitally during April 2020 and October 2020. The final sample used for the analysis contained answers from 7421 respondents. The summary statistics for variables used in the model estimation is available in Table 1.

**Table 1:** Summary statistics for variables included in final model estimations.

Variable description	Mean	Standard deviation
Socio-demographic factors		
Women indicator (1 if respondent is a woman, 0 otherwise)	0.63	0.48
Men indicator (1 if respondent is a man, 0 otherwise)	0.37	0.48
Black race indicator (1 if respondent is Black, 0 otherwise)	0.11	0.32
Asian race indicator (1 if respondent is Asian, 0 otherwise)	0.05	0.22
Household size	2.70	1.47
Age	49.5	17.4
Household residence indicator (1 if respondent rents, 0 otherwise)	0.31	0.46
Part time employment indicator (1 if respondent works part-time, 0 otherwise)	0.13	0.34
Full time employment indicator (1 if respondent works full-time, 0 otherwise)	0.45	0.50
Student status (1 if respondent is a student, 0 otherwise)	0.13	0.33
Graduate education indicator (1 if respondent has a graduate education, 0 otherwise)	0.20	0.40
No vehicle indicator (1 if respondent’s household does not own a vehicle, 0 otherwise)	0.07	0.25
Psychological and lifestyle factors		
Environmental friendliness (1 if respondent indicated their strong commitment to environmentally friendly lifestyle, 0 otherwise)	0.22	0.42
Intention to minimize pollution indicator (1 if respondent indicated their strong commitment to minimizing pollution related to transportation, 0 otherwise)	0.16	0.37
High level of life satisfaction (1 if respondent indicated high satisfaction with their life, 0 otherwise)	0.29	0.45

To capture the shift in cycling behavior, each respondent was asked about their intended cycling frequency when COVID-19 is no longer a threat, and three response categories were considered; planning to cycle more (that combined somewhat more than before and much more than before), planning to cycle less (that combined somewhat less than before and much less than before), and planning to cycle about the same amount as before the pandemic.

Using these three response categories, a random parameters multinomial logit model with heterogeneity in means and variances was estimated (no heterogeneity in the variances was detected). This approach allows the mean and variance of random parameters to be functions of explanatory variables and thus provides additional accuracy in studying unobserved heterogeneity. Researchers from the fields of travel behavior and highway safety regularly apply this statistical approach to capture unobserved effects by allowing the parameters to vary across the sample population. In the transportation field, this approach of accounting for unobserved heterogeneity, and enabling novel findings that deliver more insights into the interactions among the variables, has been successfully applied in the past^32,33^.

To estimate model that offers the best fit for the data, a function that determines the probability of either a respondent intending to cycle the same amount post pandemic, cycle less, or cycle more is defined as:

1 $$ F_{kn} = {{\varvec{\upbeta}}}_{k} {\mathbf{X}}_{kn} + \varepsilon_{kn} $$

where **X**_*kn*_ is a vector of explanatory variables that affect the probability of observation *n* being a respondent’s intended cycling frequency alternative *k*, **β**_*k*_ is a vector of estimable parameters for observation *k*, and ε_*kn*_ is a disturbance term. If the disturbance term is assumed to be generalized extreme-valued distributed, a logit model results as^34^,

2 $$ P_{n} \left( k \right) = \frac{{EXP\left[ {{{\varvec{\upbeta}}}_{k} {\mathbf{X}}_{kn} } \right]}}{{\sum\nolimits_{\forall K} {EXP\left[ {{{\varvec{\upbeta}}}_{k} {\mathbf{X}}_{kn} } \right]} }} $$

where *P*_*n*_(*k*) is the probability of the respondent *n* intended cycling behavior *k* (stay the same, increase, or decrease) when COVID-19 is no longer a threat.

To account for the possibility that one or more parameter estimates in the vector **β** may vary across respondents due to unobserved heterogeneity, a distribution of these parameters can be assumed, and Eq. (2) can be rewritten as^35^:

3 $$ {\text{P}}_{{\text{n}}}^{{}} \left( k \right) = \int {\frac{{EXP\left( {{{\varvec{\upbeta}}}_{k} {\mathbf{X}}_{kn} } \right)}}{{\sum\limits_{\forall K} {EXP\left( {{{\varvec{\upbeta}}}_{k} {\mathbf{X}}_{kn} } \right)} }}} f\left( {{{\varvec{\upbeta}}}_{k} |{\mathbf{\varphi }}_{k} } \right)d{{\varvec{\upbeta}}}_{k} $$

where, *f*(**β**_*k*_|**φ**_*k*_) is the density function of **β**_*k*_ and **φ**_*k*_ is a vector of parameters describing the mixing density function (mean and variance), and all other terms are as previously defined.

To provide more flexibility in accounting for unobserved heterogeneity, with the mixing distribution now allowing parameters to vary across observations *n*, the **β**_*kn*_ vector can be made to be a function of variables that affect its mean and variance as^33,36,37^

4 $$ {{\varvec{\upbeta}}}_{{{\text{kn}}}} = \beta_{k} { + }\Theta_{kn} {\mathbf{Z}}_{{{\text{kn}}}} + \, \sigma_{kn} EXP\left( {\Psi_{kn} {\mathbf{W}}_{kn} } \right)_{ \, } \nu_{kn} \, $$

where, β_*k*_ is the mean parameter estimate across all cycling alternatives, **Z**_*kn*_ is a vector of observation-specific explanatory variables that captures heterogeneity in the mean that affects cycling alternative *k*, **Θ**_*kn*_ is a corresponding vector of estimable parameters, **W**_*kn*_ is a vector of observation-specific explanatory variables that captures heterogeneity in the standard deviation (variance) σ_*kn*_ with corresponding parameter vector Ψ_*kn*_, and *v*_*kn*_ is a disturbance term.

The model estimation was done by simulated maximum likelihood approach and used 1000 Halton draws as they can deliver more efficient distribution of simulation draws than purely random draws^34,38^. Just like in other studies in travel behavior to achieve the most superior estimation, the normal distribution was assumed for random parameters^39,40^. Marginal effects were calculated to determine the effect of individual explanatory variables on probabilities of intended cycling frequencies. Marginal effects were averaged over all observations and are presented in Table 3. The marginal effect of an explanatory variable shows the effect that of a one-unit increase in that explanatory variable has on the response probabilities. For indicator variables (that assume values of zero or one), marginal effects will give the effect of the explanatory variable going from zero to one^35^.

## Results

### Insights into intended post pandemic cycling behavior

The largest category of the respondents included those who did not plan to change their behavior and this group represented 82 percent of the sample. The second largest category (13.5 percent of the sample, or 1002 observations), was the group, where the respondents stated that they were planning to cycle more (somewhat more or much more than before the pandemic). Only 4 percent of the respondents stated that they were planning to cycle less (somewhat less than or much less than before). This rather large difference in percentage points between the respondents who intended to cycle more (13.5 percent) and those who intended to cycle less (4 percent) likely reflects the fact that the COVID-19 pandemic provided an opportunity to experiment with new transportation modes such as cycling, and this experimentation may have long-lasting impact on mobility behavior. When asked about the reasons for their intention to increase cycling, the majority of the respondents stated that they realized they liked biking, and this was followed by their expectation to bike more in their neighborhoods (Table 2). The increase in the intended cycling frequency in a respondent’s neighborhood is likely dictated by the changes to the infrastructure or right-of-way in their immediate community in response to the COVID-19 pandemic^19^.

**Table 2:** Reasons for the intended increase in cycling post COVID-19 pandemic (n = 1002).

Reason for increasing cycling	Number of respondents selecting this option^a^
I realized I really like biking	555
I expect to bike more in my neighborhood	479
I realized biking is an inexpensive way to get around	302
I expect to use biking to replace trips by other means of transport	203
I realized biking is fast	182
I bought a bike	178
I expect my city to make biking safer	173

^a^Multiple responses per individual were allowed.

The mixed logit model with heterogeneity in the means of random parameters estimation results (Table 3) will provide additional insights into the variables playing a role in intended cycling frequency post COVID-19 pandemic and show that both socio-demographic and various lifestyle choices and preferences were statistically significant.

**Table 3:** Random parameters logit model with heterogeneity in the mean of random parameter on intention to cycle the same amount [S], less [L] or more [M] when COVID-19 pandemic is no longer a threat.

Variable description	Estimated parameter	t-statistic		Marginal effects		
				Cycle the same [S]	Cycle less [L]	Cycle more [M]
Constant [L]	− 3.62	− 28.03				
Constant [M]	− 1.36	− 6.64				
Socio-demographic factors						
Random parameters						
Women indicator (1 if respondent is a woman, 0 otherwise) [M] (Standard deviation of parameter distribution)	− 1.47 (1.95)	− 3.68 (4.40)		− 0 .0118	− 0.0005	0.0124
Heterogeneity in the mean of the random parameter						
Women indicator: no vehicle (1 if respondent’s household does not own a vehicle, 0 otherwise)	0.49	1.91				
Fixed parameters						
Men indicator (1 if respondent is a man, 0 otherwise) [L]	0.34	2.96		− 0.0050	0.0060	− 0.0010
Black race indicator (1 if respondent is Black, 0 otherwise) [L]	0.49	3.09		− 0.0027	0.032	− 0.0004
Asian race indicator (1 if respondent is Asian, 0 otherwise) [M]	− 0.33	− 1.74		0.0015	0.0001	− 0.0016
Household size [L]	0.17	5.23		− 0.0187	0.0214	− 0.0027
Age [M]	− 0.02	− 5.81		0.0719	0.0040	− 0.0759
Household residence indicator (1 if respondent rents, 0 otherwise) [M]	− 0.28	− 2.80		0.0070	0.0004	− 0.0074
Part time employment indicator (1 if respondent works part-time, 0 otherwise) [M]	0.36	2.55		− 0.0045	− 0.0003	0.0047
Full time employment indicator (1 if respondent works full-time, 0 otherwise) [M]	0.41	2.55		− 0.0195	− 0.0012	0.0206
Student status (1 if respondent is a student, 0 otherwise) [M]	0.84	6.74		− 0.0148	− 0.0010	0.0158
Graduate education indicator (1 if respondent has a graduate education, 0 otherwise) [M]	0.28	2.81		− 0.0060	− 0.0004	0.0063
Psychological and lifestyle factors						
Environmental friendliness (1 if respondent indicated their strong commitment to environmentally friendly lifestyle, 0 otherwise) [M]	0.29	2.64		− 0.0073	− 0.0004	0.0078
Intention to minimize pollution indicator (1 if respondent indicated their strong commitment to minimizing pollution related to transportation, 0 otherwise) [M]	0.62	5.20		− 0.0124	− 0.0008	0.0132
High level of life satisfaction (1 if respondent indicated high satisfaction with their life, 0 otherwise) [M]	0.23	2.54		− 0.0066	− 0.0004	0.0070
Number of observations	7421					
Log likelihood at zero, LL(0)	− 8152.80					
Log likelihood at convergence, LL(β)	− 4018.25					
ρ^2^ = 1 – LL(β)/LL(0)	0.51					

The effect of each independent variable on the probability of intended cycling frequency is specified by the marginal effects and notations [S], [L], [M].

### Model estimation results: Socio-demographic factors

Turning to the model estimation results, gender was found to be a significant factor in determining respondents’ cycling behavior. This finding is not novel as it has been confirmed by a large body of research^41–43^. The nuance that the current analysis offers, however, is that the effect of the women indicator variable is heterogeneous across women respondents, as indicated by the statistically significant standard deviation of the random parameter shown in Table 3. Furthermore, Table 3 shows that women respondents without a vehicle in their households had an increase in their mean, implying they were more likely to indicate that they intend to cycle more post the pandemic than their vehicle-owning female counterparts. Respondents who were men, on the other hand, were found to have a higher probability of intending to cycle less (as indicated by the marginal effect). The gender differences present in intended cycling frequency likely reflect a much broader issue around gender and mobility.

Particularly in the light of the fact that, globally, women have been found to cycle less than men^41,44^, the outcome of the current analysis suggests an ongoing shift and possible increase in cycling by women post pandemic.

Disparities in cycling behavior are not only linked to gender but are also present among minority groups. Former research argues that the COVID-19 pandemic has created opportunities for cities to close streets to automobile traffic to promote public health and other civic objectives. Although these interventions promised numerous benefits, neighborhood activists and scholars of color suggest they can perpetuate structurally racist inequities^45^. Findings herein, to some extent, confirm these concerns as the model results indicate that the respondents who were Black and those who were Asian will not cycle more but rather less. Turning to the abovementioned results regarding gender and race, and intended post COVID-19 cycling frequency, it is essential to broaden the conversation and emphasize that it is rarely a single element that determines mobility behavior, and the totality of distributional justice, accessibility, and safety, and their varied impacts on different socio-demographic groups, must be given full consideration^45^.

Respondent’s age and their household size were also found statistically significant variables suggesting as the individual gets older, they will have a lower probability of intending to cycle more (as indicated by the marginal effect equal to − 0.0759 in Table 3), whereas as their household size increases, they will have a higher probability of intending to cycle less. Respondents who rent their residence were found to have a lower probability of intending to cycle more post COVID-19 pandemic. The findings relating to age are not surprising, in a sense that with age, the propensity to engage in active mobility modes decreases and the probability of injury increases^46^. Nevertheless, the insights on the household size and residential renting and their impact on intended cycling frequency are interesting, as they capture the complexity of human behavior with regard to personal mobility. Particularly, the impact of increasing household size and its role in increasing the probability of intending to cycle less, likely captures the number of children in households, which has been confirmed to have a strong relationship to cycling behavior of adults^22,31^.

Interestingly, both groups of workers in the sample (those who work full time, and those who work part time), were found to have a 0.0047 and 0.0206 higher probability of intending to cycle more after the pandemic (as indicated by the marginal effects in Table 3). Because cycling has become a much more appealing transportation alternative to many, especially during the peak of COVID-19 cases, some workers throughout that time found themselves using this mode^47^. Combining these results with the findings from Table 2, which indicate that a significant number of respondents who indicated an intended increase in cycling, marked ‘I realized I really like biking’ as one of their reasons, provides an explanation that experiencing a particular phenomenon can drastically change how one feels about it, and whether they will adopt a new behavior in the long term^32^.

The last set of variables relating to the socio-demographic factors includes respondents who were students and those with a graduate level education. Both groups had a higher probability of intending to cycle more post COVID-19 pandemic. This is predominantly important in the case of students, who are still forming their long-term mobility behaviors that they will carry into their adult lives. The fact that they had a 0.0158 higher probability of intending to cycle more as indicated by marginal effects, offers an attractive opportunity for the universities to continue to build momentum around cycling. Even before the pandemic, it was not unusual for university campuses and college towns to invest in micromobility modes and networks to mitigate congestion and emissions as well as nudge the students toward physical activity through active transportation^48^.

### Model estimation results: The psychology of cycling

In addition to the standard socio-demographic variables that are often considered in travel behavior research, this study explored a set of lifestyle and psychological variables that likely capture a wide variety of unobserved factors found to dictate how people behave and what choices they make^49^.

Environmental friendliness reflected in respondents’ self-reported commitment to live environmentally friendly lifestyle as well as respondent’s strong commitment to minimizing pollution related to transportation were both statistically significant factors in the model. Respondents from these two groups were found to have a higher probability of intending to cycle more post pandemic as indicated by the marginal effects in Table 3 (0.0078 for the respondents committed to environmentally friendly lifestyle and 0.0132 for the respondents committed to minimizing pollution from transport). Interestingly, the magnitude of the probability of the respondents who are committed to minimizing pollution coming from transport is much larger than of the ones committed to the environmentally friendly lifestyle, which suggests opportunities to emphasize the role of transportation in promoting sustainable lifestyles.

Lastly, respondents who indicated a high satisfaction with their life had a slightly 0.007 higher probability of intending to cycle more than those who did not indicate such satisfaction. This finding is consistent with the literature arguing that life satisfaction is directly and indirectly related to satisfaction with travel^50^. Abou-Zeid and Ben-Akiva^51^ found that activity happiness and travel satisfaction are strongly correlated with activity participation with the greater the activity happiness and the greater the satisfaction with travel to the activity, the higher is the propensity of conducting the activity. Perhaps, what is the most interesting about this result is the opportunity to trigger further discussion about how cycling can be leveraged to support public health and well-being on a systemic level.

## Discussion

The findings do not only deliver insights into intended cycling frequency post COVID-19 but also stress out the importance of heterogeneous cycling behavior among different groups. Particularly considering the ongoing climate crisis and the efforts to decarbonize transportation, as well as the need to shift from auto-centric to human-centered designs, understanding which factors are critical in the further uptake of cycling is essential for planning urban cores. Although, systemic changes in how people travel are difficult to achieve, some cities around the world were able to gain momentum and overtime transform their downtowns. Even before the pandemic, cycling infrastructure expenditure that was supported by policies targeting less sustainable modes, was found to be associated with more cycling among commuters^52^. Nonetheless, work herein looks beyond the infrastructure and examines how different groups of people have responded to COVID-19 measures and how that affected their intended cycling frequency. Considering the complexity of human behavior, fully grasping travel preferences, and supporting the findings with statistically significant results is not an easy proposition. Because each person has arguably an unlimited number of factors impacting their behavior, identifying one observable factor, and combining it with other identified variables allowed a clearer profile of a cyclist who intends to cycle more. Looking beyond the system and focusing on individual travelers allows a broadening of the ongoing cycling dialogue by examining this shift through multiple lenses including environmental justice, equal access, gender equality, and various psychological factors.

Although cycling is an inexpensive and accessible mode of travel, its adoption does not happen uniformly as it is often tied to the built environment, social acceptance, perceived safety, and cycling culture. Savan et al.^27^ identified strategic population segmentation as one of the key approaches to promote cycling. The authors argued that the importance of recognizing the needs of individual preferences is crucial to create successful cycling policies. The model findings herein, to some extent, deliver this information, as they were able to capture those differences in individual behaviors and preferences. Particularly interesting is the fact, that workers (full time and part time) have a higher probability of intending to cycle more after the pandemic. This result points towards the significance of evaluating cycling in the context of transportation as opposed to recreation. Supporting workers and creating policies that would further codify the shift triggered by the pandemic, could provide long term benefits in decreasing congestion, minimizing emissions, and increasing physical activity. As former research has shown, personalized travel programs usually involve strategically targeted information, events, and incentives to achieve most optimal results in causing mode shift^53,54^. Others argue that cycling promotion programs should take advantage of life course transition periods as opportunities to target the intended behavior change^55^. With that said, the disruption caused by COVID-19 pandemic serves as a perfect opportunity to capture groups who intend to cycle more. Policies and practices are needed to reduce the distance travelled by vehicles, support active travel like cycling and walking, public transit, and compact development to nurture this global transition^56^.

However, there is also another side of the story that needs to be told. Interventions to close streets to auto traffic and promote open streets to allow walking and cycling have promised numerous benefits, but some have indicated that these could preserve structurally racist inequities^45,57^. It is without a question that the pandemic has opened a new window of opportunity for original solutions untethered to former constraints, but also brought potential paradoxes emerging from open street implementation. Such nuances are important to consider in the light of the findings of this study, which concluded that people who are Black and Asian have a lower probability of intending to cycle after the pandemic. Instances of environmental injustices include both, the concentration of environmental amenities in Whiter communities and the concentration of environmental hazards in BIPOC (Black, Indigenous and People of Color) communities^45^. Therefore, it is essential to bring such considerations to the forefront in the conversations and advocacy relating to cycling, urban design, and access.

The results of this study can be also used to understand the barriers to cycling and as bases for policy formation. Given the potential reduction in greenhouse gas emissions reduction that cycling offers, the cycling mode merits more consideration, investment, and political support if Paris Agreement goals are going to be met.

Lastly, looking from a system-level perspective, achieving radical reductions in car use will require deep carbon reductions in all locations and a reduction in car use that is reflected in national average per capita statistics, not just in a select few inner-city locations^58^. To target emission reductions, alternatives such as e-bikes have shown promise, with research arguing that e-bikes could play a major role in carbon reduction of land-based transport and offer even larger CO_2_ savings, especially when considering a mode switch from a personal vehicle to e-bike^59,60^.

## Data Availability

The datasets generated and analyzed during the current study are available in the COVID-19 and the Future Survey repository, https://covidfuture.org/data/.
